# Assessing the impact of turbulent kinetic energy boundary conditions on turbulent flow simulations using computational fluid dynamics

**DOI:** 10.1038/s41598-023-41324-w

**Published:** 2023-09-05

**Authors:** Eui Cheol Jung, Gyu-Han Lee, Eun Bo Shim, Hojin Ha

**Affiliations:** 1 Kangwon Institute of Inclusive Technology, Kangwon National University, 1, Kangwondaehak-Gil, Chuncheon, 24341 Republic of Korea; 2 Institute of Medical Devices, Kangwon National University, 1, Kangwondaehak-Gil, Chuncheon, 24341 Republic of Korea; 3 Department of Mechanical and Biomedical Engineering, Kangwon National University, 1, Kangwondaehak-Gil, Chuncheon, 24341 Republic of Korea

**Keywords:** Engineering, Biomedical engineering

## Abstract

Computational fluid dynamics has been widely used to study hemodynamics, but accurately determining boundary conditions for turbulent blood flow remains challenging. This study aims to investigate the effect of patient-specific turbulence boundary conditions on the accuracy of turbulent flow simulation. Using a stenosis model with 50% severity in diameter, the post-stenosis turbulence flow region was simulated with different planes to obtain inlet boundary conditions and simulate downstream flows. The errors of simulated flow fields obtained with turbulence kinetic energy (TKE) boundary data and arbitrary turbulence intensity were compared. Additionally, the study tested various TKE data resolutions and noise levels to simulate experimental environments. The mean absolute error of velocity and TKE was investigated with various turbulence intensities and TKE mapping. While voxel size and signal-to-noise ratio of the TKE data affected the results, simulation with SNR > 5 and voxel size < 10% resulted in better accuracy than simulations with turbulence intensities. The simulation with appropriate TKE boundary data resulted in a more accurate velocity and turbulence field than those with arbitrary turbulence intensity boundary conditions. The study demonstrated the potential improvement of turbulent blood flow simulation with patient-specific turbulence boundary conditions, which can be obtained from recent measurement techniques.

## Introduction

Hemodynamic assessment is important for the diagnosis, treatment, and management of cardiovascular diseases (CVDs). The severity of the aortic stenosis is assessed with the flow velocity and corresponding pressure gradient^1–3^. The high and oscillatory wall shear stress has been correlated to the aortic dilatation and possible development of the aneurysm^4,5^. The elevated level of the turbulent flow also suggests the aortic stenosis, aortic coarctation, valvular dysfunction and graft kinking^6–8^. The hemodynamics has been also monitored during the interventional treatment. The flow velocity and corresponding WSS has been checked to confirm the effect of the flow diverter and endovascular coil^9,10^. In the case of aortic dissection, the flow rate and velocity though the true and false lumens are studied to predict the prognosis of the disease and make a plan for the stent implantation or the fenestration point^11,12^.

Computational fluid dynamic (CFD) simulation is a widely used method for investigating hemodynamics. Recently, with advances in computing power, medical image acquisition techniques, and processing methods, the patient-specific CFD has enabled non-invasive hemodynamics assessment and diagnosis, replacing the conventional invasive techniques. In addition, CFD can play a complementary role in conventional blood flow measurements such as Doppler echocardiography and phase-contrast magnetic resonance imaging (MRI), which usually have a limited spatial–temporal resolution. For example, various studies have been trying to replace the invasive fractional flow reserve measurement by simulating the coronary blood flow and assessing the stenosis severity based on the patient’s CT images^13–17^. In addition, a study that used intravascular ultrasound(IVUS) to create a three-dimensional vessel model and CFD-based simulation to analyze blood flow showed high quality simulation results based on a precise vessel model^18,19^. A patient-specific CFD using Doppler echocardiography and phase-contrast MRI also showed that patient-specific computational modeling could provide complimentary diagnostic insight for more rigorous assessment of present hemodynamics^20^.

Specifying an appropriate boundary condition is one of the most important steps in CFD to obtain accurate results. Blood flow rate, pressure, and microvascular resistance at the inlet and outlet should be directly measured or numerically modeled. Coronary flow is frequently estimated to be proportional to the mass of left ventricle myocardium or the volume variation in the aorta^21,22^. When the direct measurement of the blood flow is applicable, the simulations using the inlet velocity and corresponding flow rate from Doppler echocardiography and phase-contrast MRI enhances the accuracy and reliability of the computation^23,24^.

Physiological blood flow develops turbulence in various vascular regions. In particular, turbulence is often observed around stenotic, curved and branched blood vessels. The turbulence exhibits highly chaotic velocity and pressure fluctuation typically ranging from micrometers to meters and microseconds to seconds^25^. The elevated level of turbulence is related to the unnecessary energy dissipation of the blood flow and endothelial cell dysfunction due to the chaotic nature of turbulence^26–28^. In addition, turbulent blood flow causes blood cell damage and promotes thrombus formation by activating platelets in the blood^29^. Therefore, accurate depiction of the turbulence nature of the blood flow is important to understand the relationship between the hemodynamics and the development of CVDs.

Despite the possible mechano-physiological role of turbulent blood flow, the accurate simulation of turbulent blood flow is yet to be realized. First, for more accurate turbulence simulation results, higher space and time resolutions are required, thereby increasing the computing power and also simulation time. Second, it is still difficult to experimentally obtain an appropriate turbulent flow boundary condition for in-vivo subjects. Conventional medical imaging such as Doppler echocardiography and phase-contrast MRI only provides peak velocity from the overlapping ultrasound signals or phase-averaged velocity from the multiple MRI echo signals^30,31^. Therefore, the previous studies on the turbulent blood flow usually assumed that the inlet flow is laminar or simple turbulence model was used based on the turbulence intensity^16,32,33^.

Recently, 4D flow MRI has been developed and used to obtain three-dimensional velocities and also Reynolds stress. A 4D flow MRI was developed to measure blood flow that changes over time in a three-dimensional space based on the MRI sensitivity^8,34,35^. Dyverfeldt et al. proposed a TKE measurement method based on the relation between the turbulent velocity produced inside the voxel and the MRI image magnitude measured in the 4D flow MRI^36^. Several in-vitro and also in-vivo studies have demonstrated that 4D flow MRI can provide not only the velocity but also turbulence parameters^37^. This advances in the non-invasive imaging technique provides experimental measurements of turbulent flow boundary data for the patient-specific CFD.

We hypothesized that applying turbulence boundary conditions by referring to 4D flow MRI measurements would result in better simulations than an arbitrary choice of turbulence intensity. Therefore, this study aimed to investigate the effect of the turbulence boundary condition on the accuracy of the blood flow simulation. In particular, the effect of arbitrary turbulent intensity was compared with the ground truth. In addition, simulations with TKE data given for the turbulence boundary condition were carried out to investigate the influence of TKE measurements from 4D flow MRI on the results. The possible effects of measurement noise and resolution from the TKE measurements were also analyzed.

## Results

### Comparison of full-model and sub-models with accurate TKE boundary conditions

**Figure 1:**
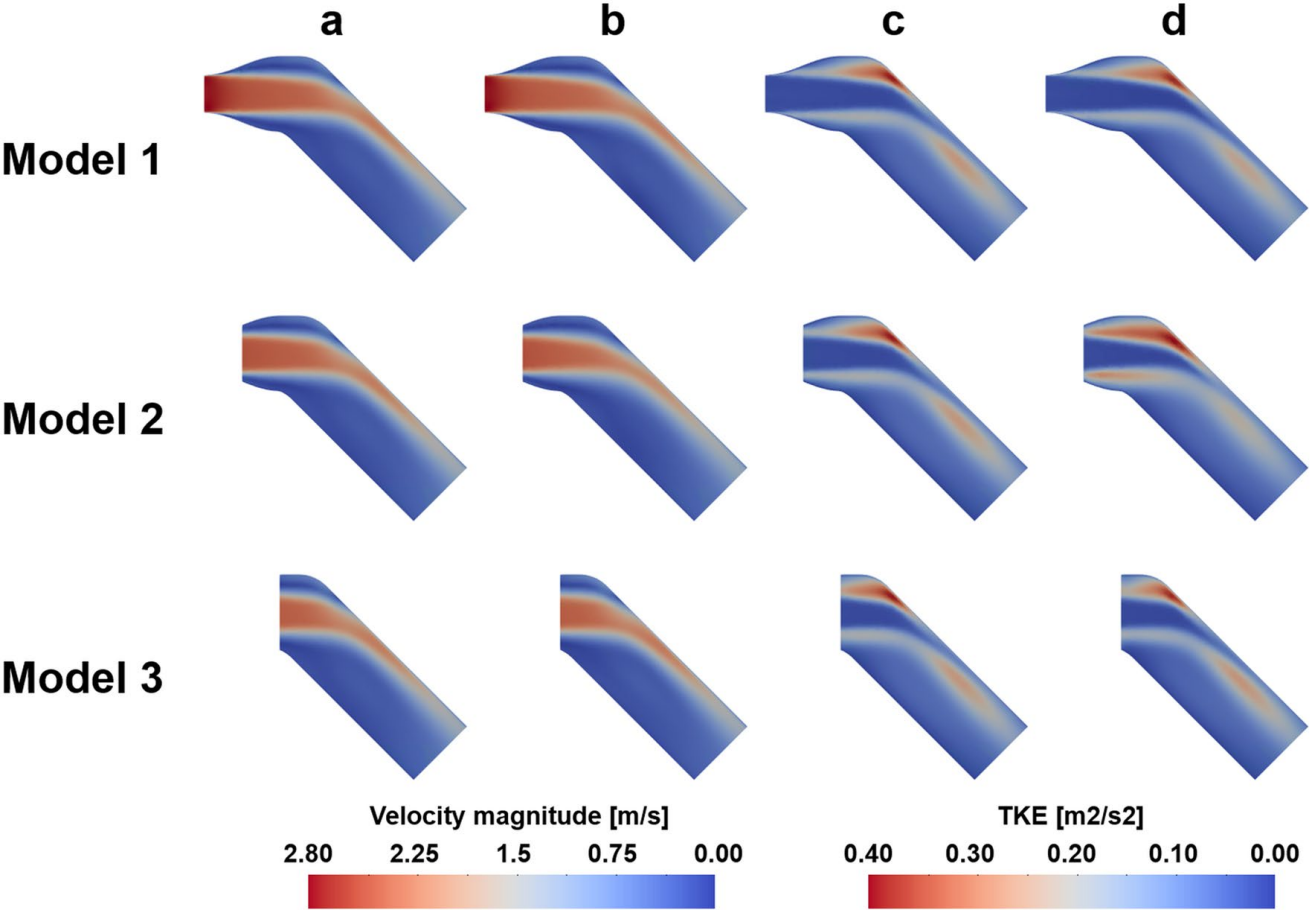
Verification of velocity and TKE field on the post-stenosis; (**a**) Ground-truth velocity, (**b**) Simulated velocity with TKE boundary data, (**c**) Ground-truth TKE, (**d**) Simulated TKE with TKE boundary data.

The three sub-models out of the original full-model were extracted and the flow fields are simulated by using the velocity and TKE boundary conditions taken from the full-model flow data. The results demonstrated that the sub-model simulations with an accurate boundary conditions successfully reconstruct the turbulent jet flow as shown from the full-model (Fig. 1 and Table 1).

**Table 1:** MAE of velocity magnitude and TKE between the result of the TKE mapping inlet boundary condition and the ground truth.

	Model 1	Model 2	Model 3
Velocity magnitude (m/s)	0.0407	0.0592	0.0192
TKE (m^2^/s^2^)	0.0062	0.0121	0.0043

The results of the velocity magnitude and TKE field in the full-model and sub-models showed qualitatively similar results. The mean absolute error (MAE) of TKE in Models 1, 2 and 3 were 0.005, 0.012 and 0.004 m^2^/s^2^, respectively, while MAE of velocity were 0.035, 0.051 and 0.027 m/s, respectively. The maximum errors of TKE for Models 1, 2, and 3 are 0.097, 0.143 and 0.075 m^2^/s^2^, respectively, while the maximum errors of velocity are 0.883, 0.359 and 0.376 m/s, respectively.

### The effect of turbulent intensity on the velocity and TKE distribution

**Figure 2:**
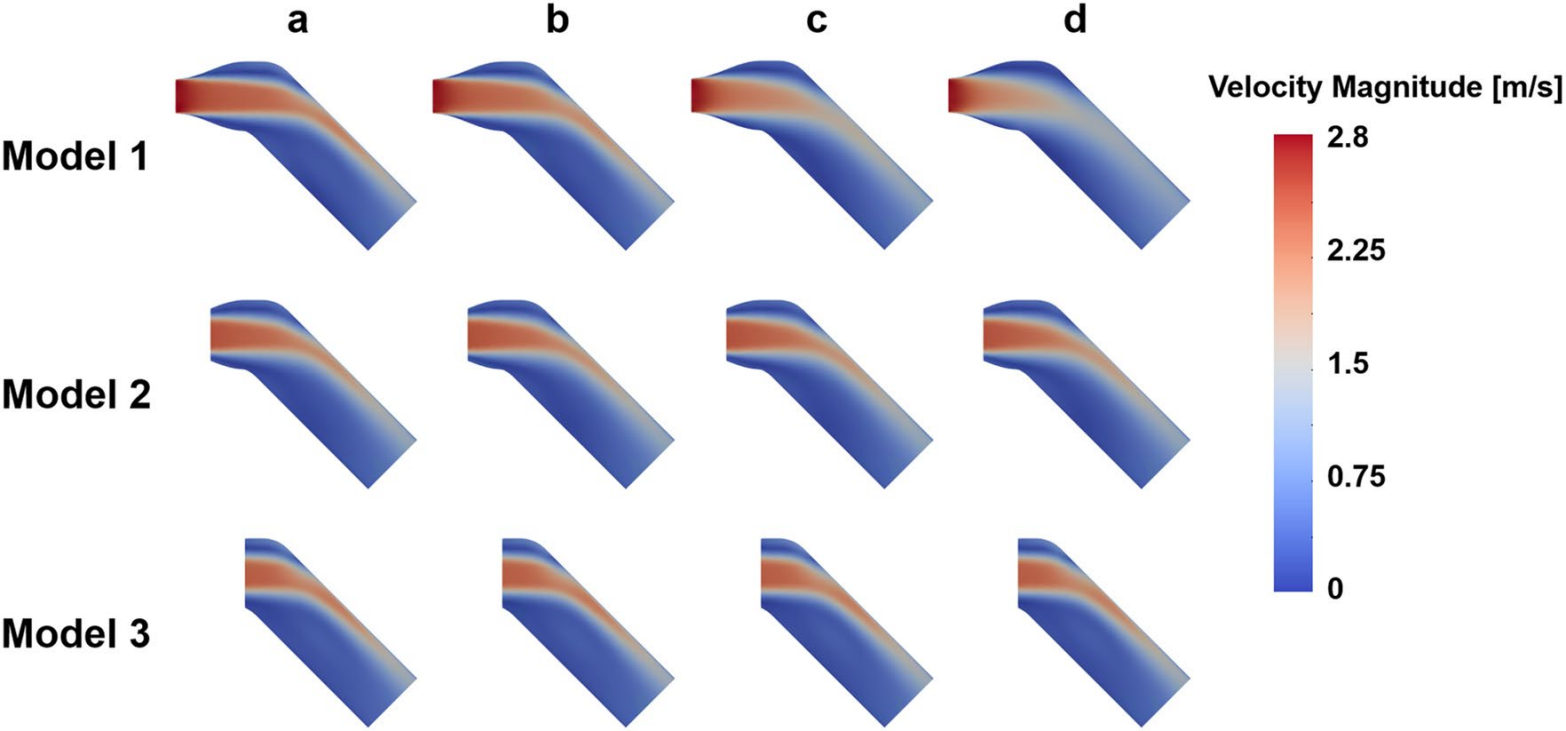
Effect of turbulent intensity on the post-stenosis velocity field; (**a**) TKE mapping, (**b**) turbulent intensity 5%, (**c**) turbulent intensity 10%, (**d**) turbulent intensity 15%

**Figure 3:**
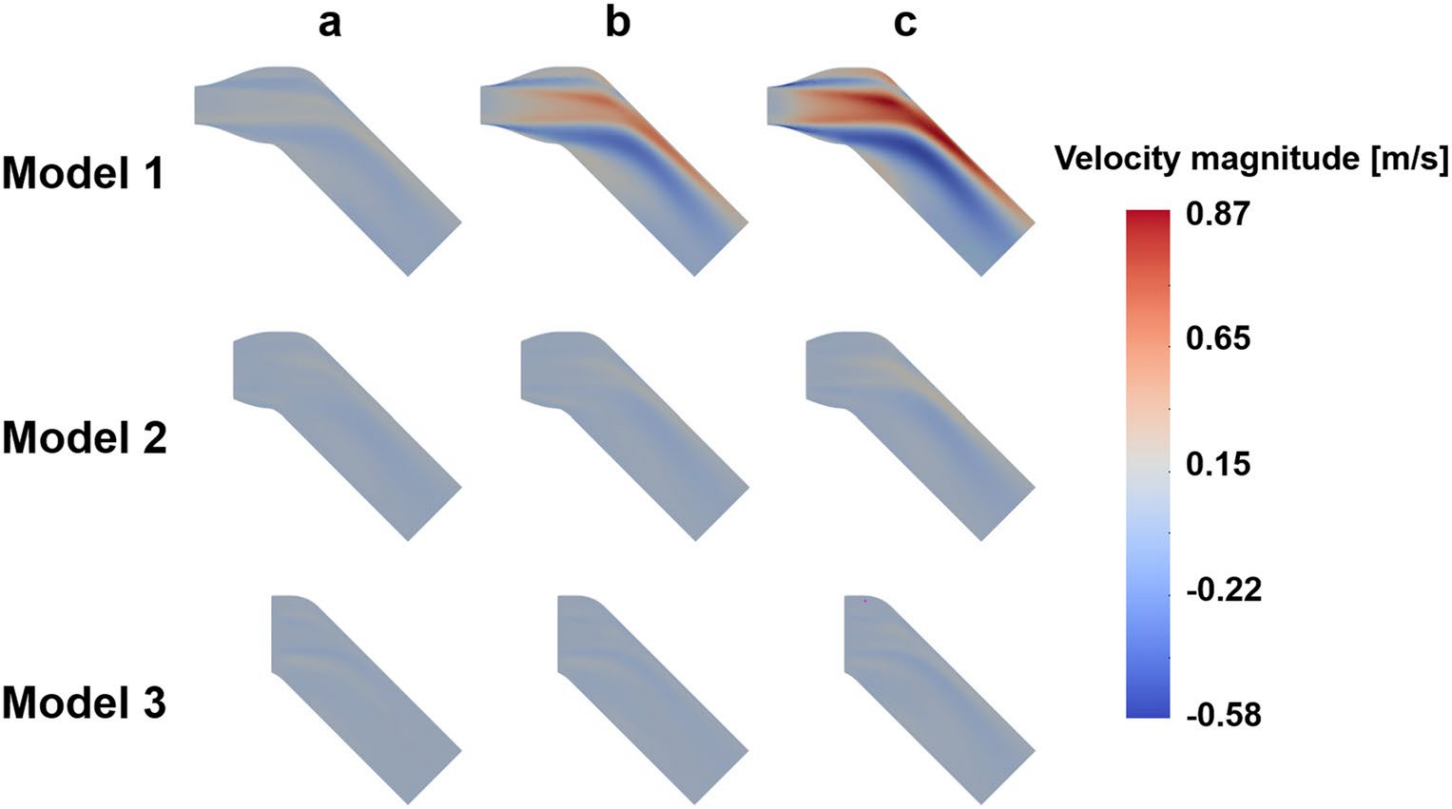
Velocity difference for different turbulent intensity; (**a**) turbulent intensity 5%, (**b**) turbulent intensity 10%, (**c**) turbulent intensity 15%

**Figure 4:**
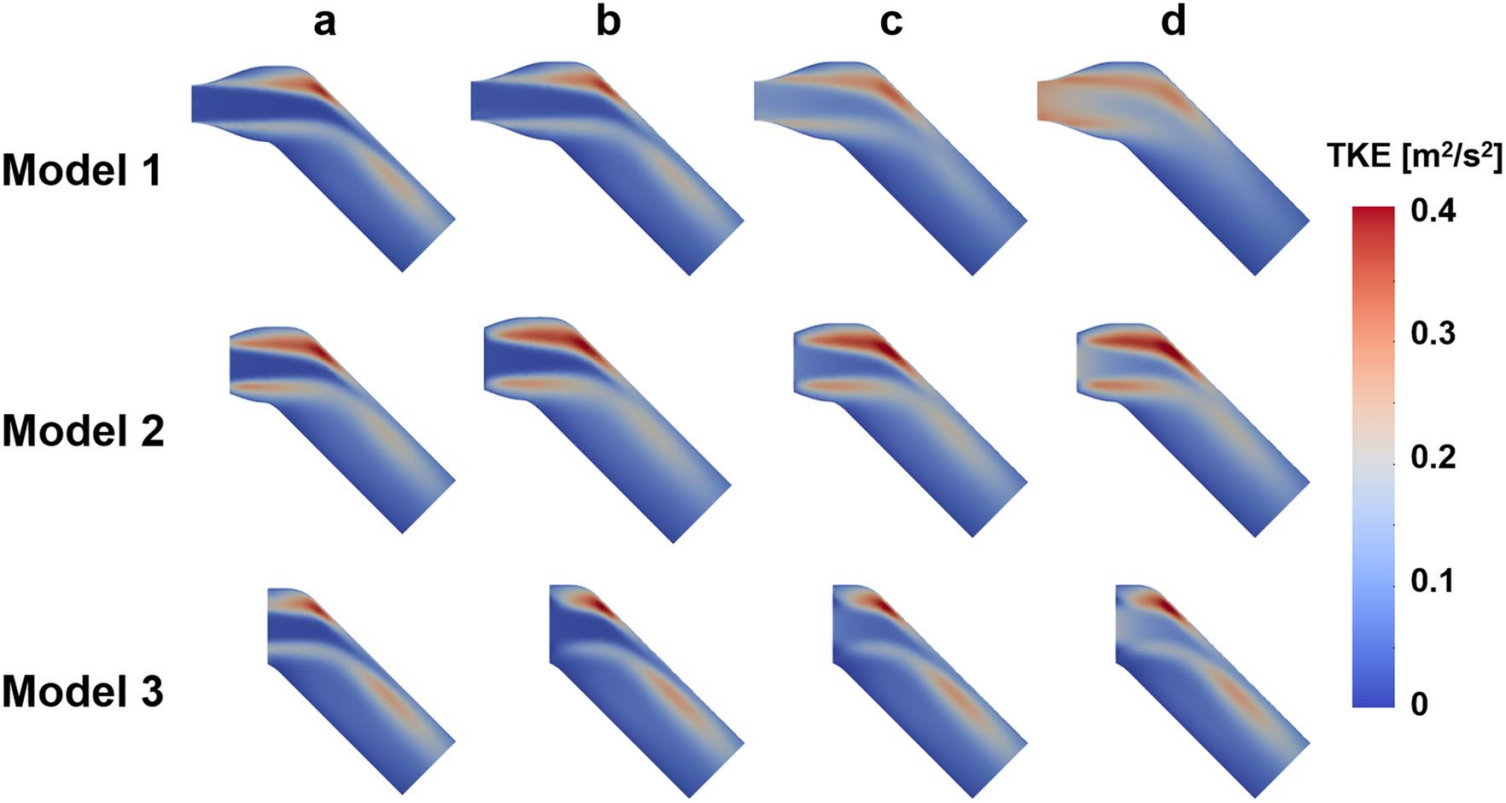
Effect of turbulent intensity on the post-stenosis TKE field; (**a**) TKE mapping, (**b**) turbulent intensity 5%, (**c**) turbulent intensity 10%, (**d**) turbulent intensity 15%

**Figure 5:**
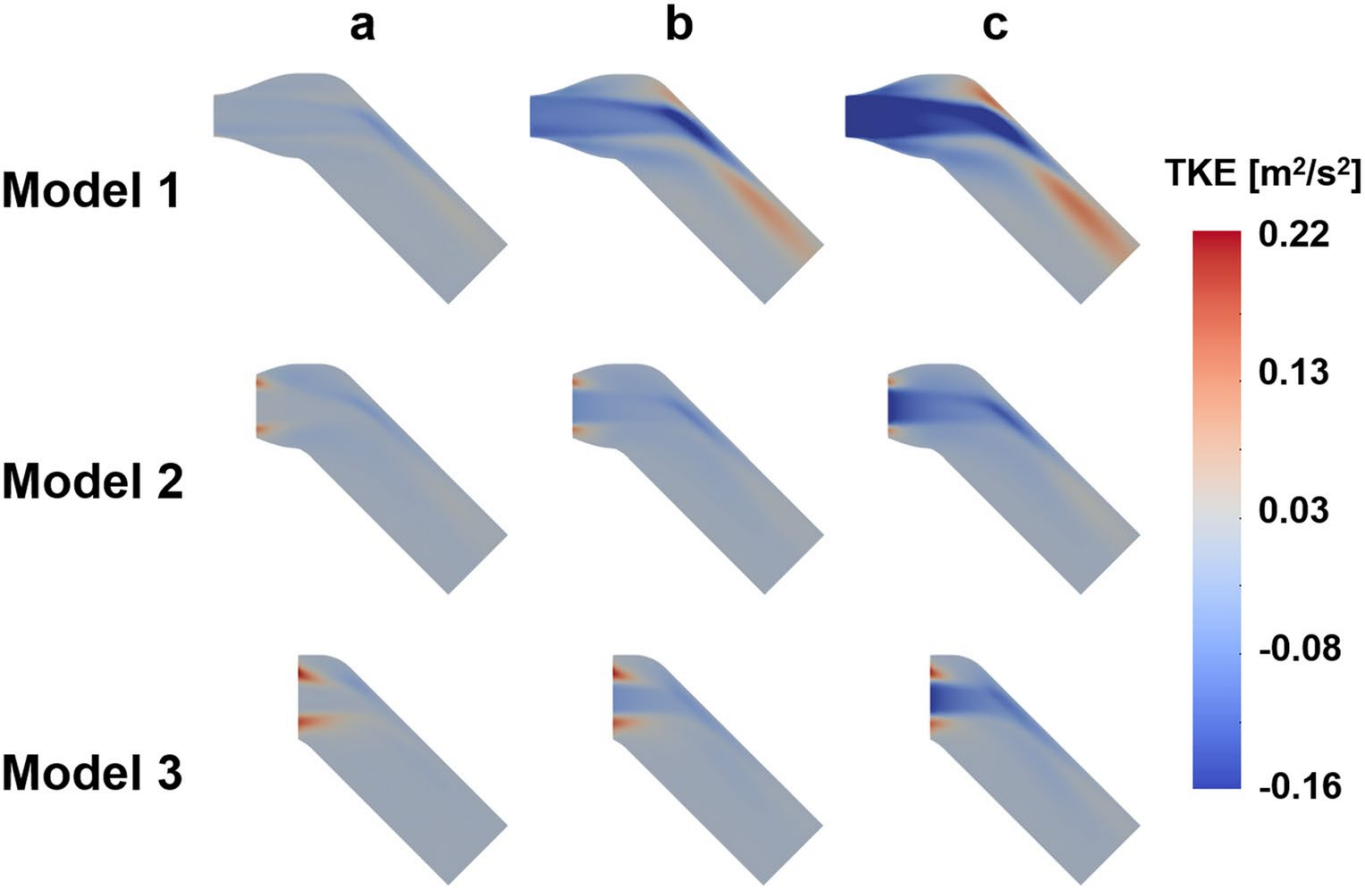
TKE difference for different turbulent intensity; (**a**) turbulent intensity 5%, (**b**) turbulent intensity 10%, (**c**) turbulent intensity 15%

**Figure 6:**
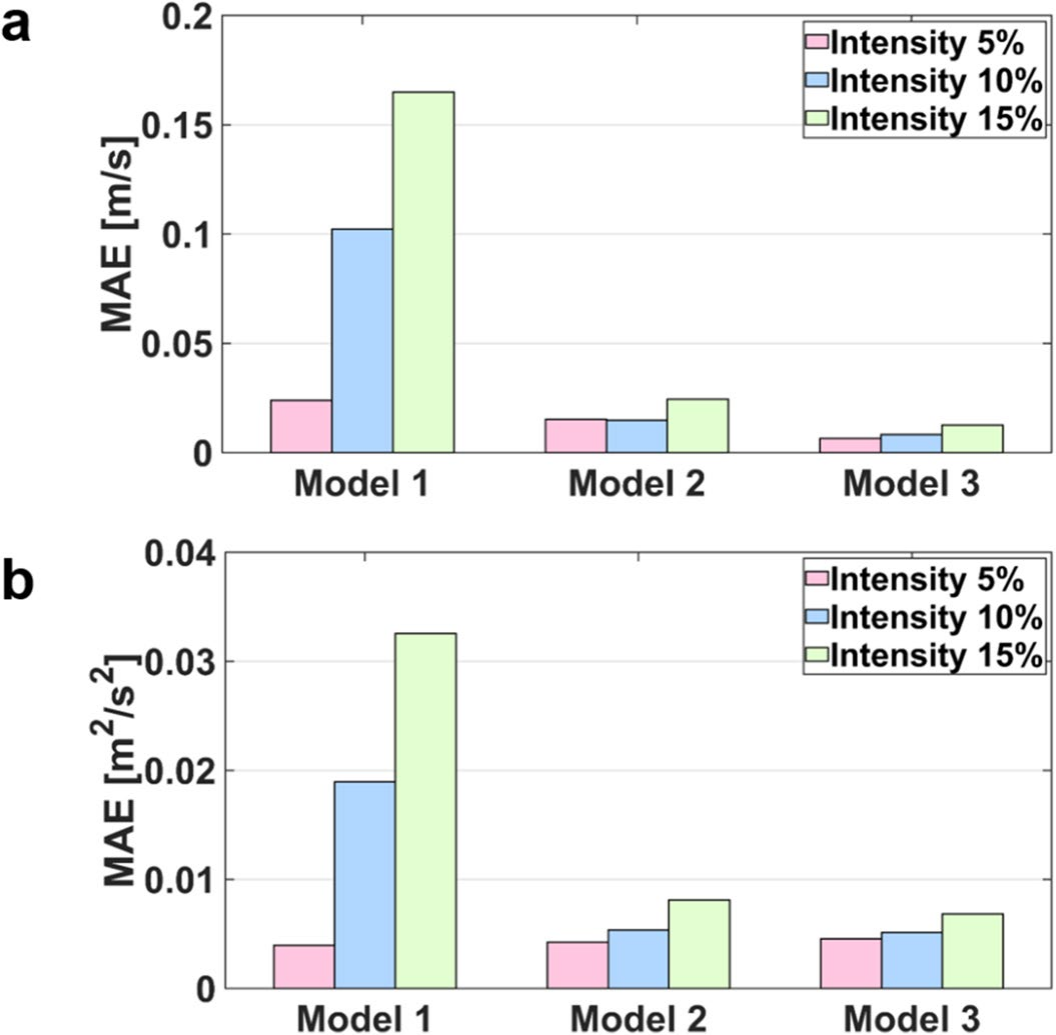
MAE of velocity magnitude and TKE compared with result of TKE mapping and turbulent intensity inlet boundary condition at the ROI.

The velocity field with three different turbulent intensities are compared with the ground-truth (Figs. 2, 3, 4, 5, 6). In general, the discrepancy of the velocity and TKE increases with the turbulent intensity. While the velocity discrepancy increases with the turbulent intensity, the velocity error was relatively minor regardless of the sub-models and the turbulent intensities. The highest MAE of velocity were 0.165 m/s at the intensity of 15% and the Model 1, which corresponds to 5.8% of the maximum velocity magnitude. The lowest MAE of velocity were 0.007 m/s at the intensity of 5% and the Model 3, which corresponds to 0.3% of the maximum velocity magnitude.

The TKE discrepancy also increases with the turbulent intensity. The highest MAE of TKE were 0.033 m^2^/s^2^ at the intensity of 15% and the Model 1, which corresponds to 9.7% of the maximum TKE magnitude. The lowest MAE of TKE were 0.004 m^2^/s^2^ at the intensity of 5% and the Model 2, which corresponds to 0.9% of the maximum TKE magnitude.

### The effect of the noise and resolution of the TKE boundary data

**Figure 7:**
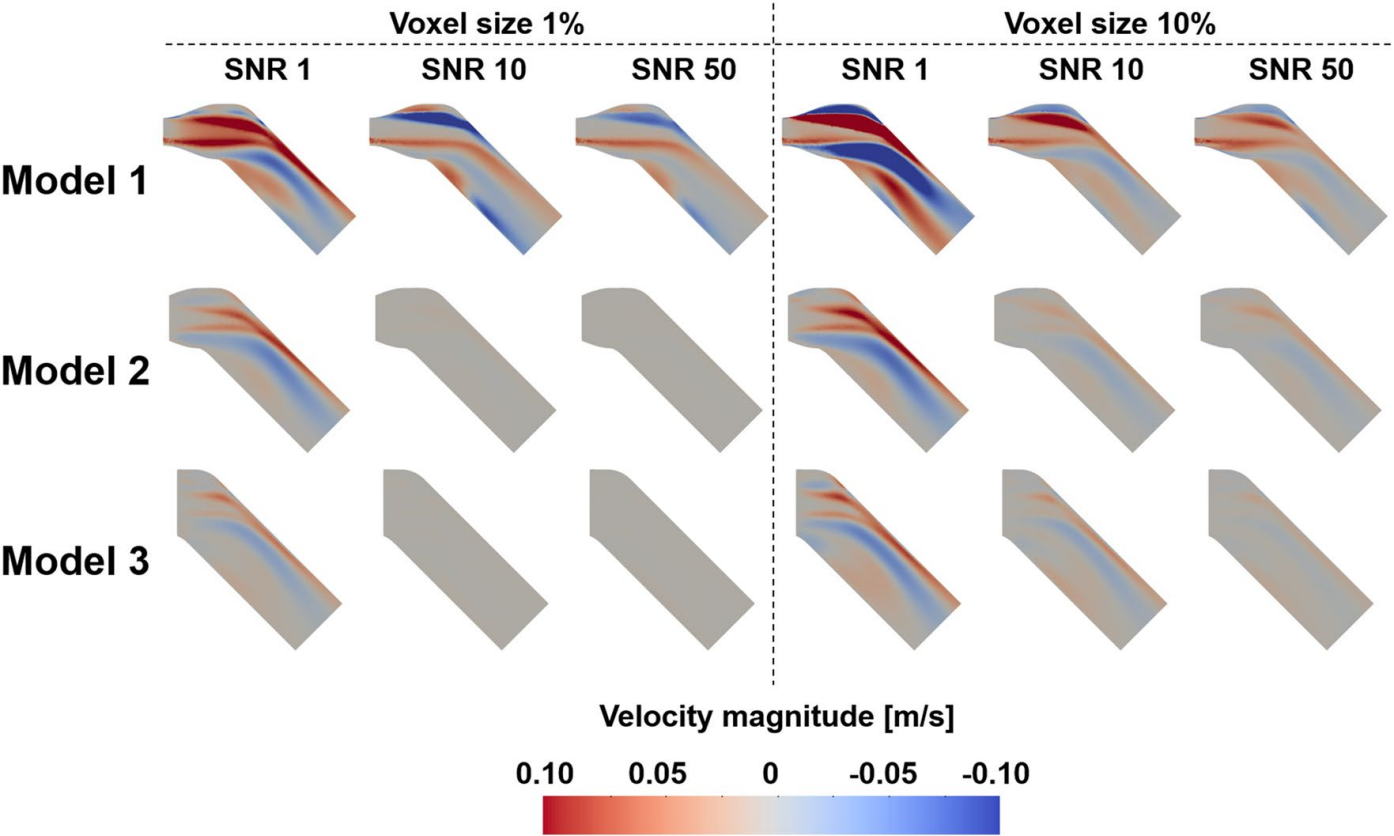
Effect of TKE resolution and noise level for the velocity magnitude difference; compared with the ground-truth.

**Figure 8:**
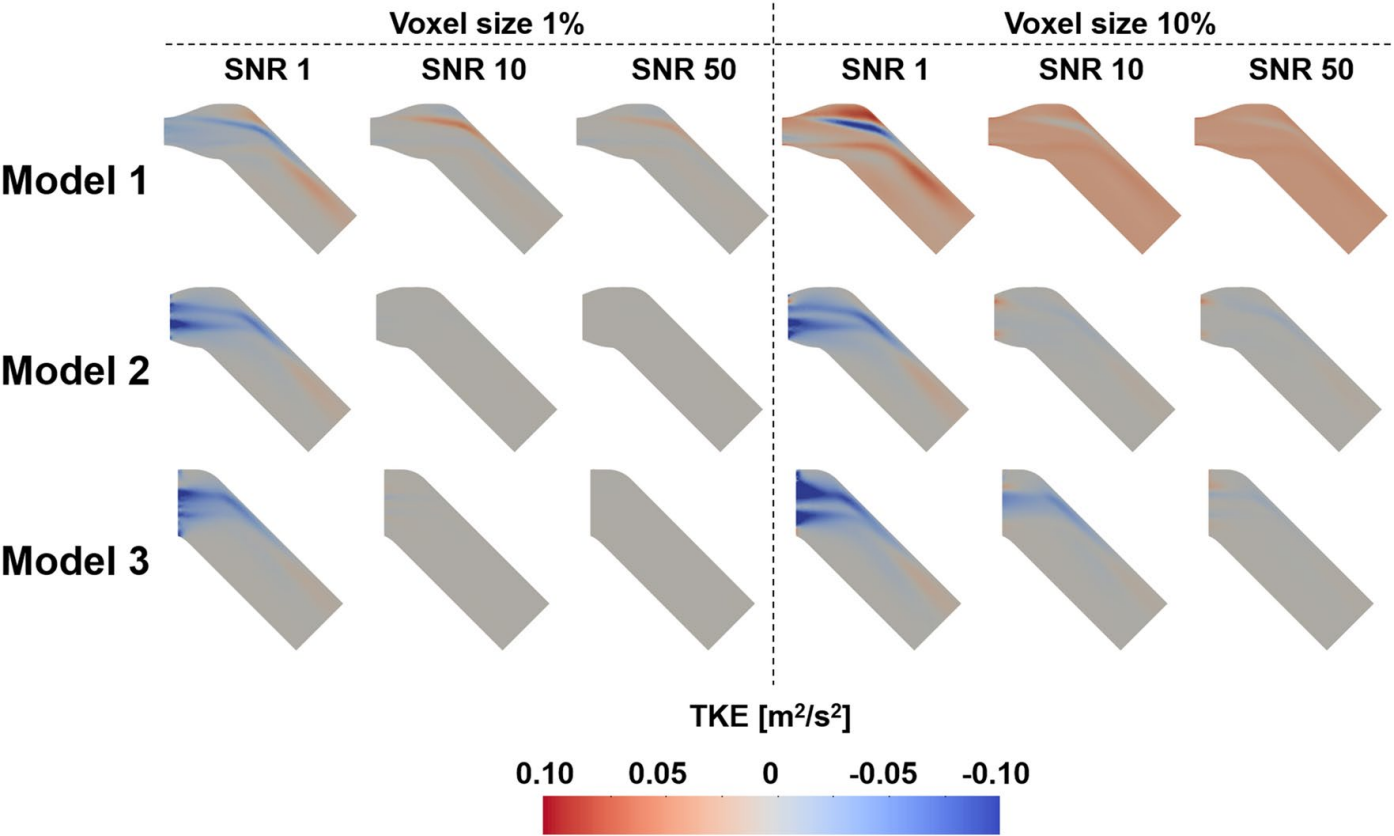
Effect of TKE resolution and noise level for the TKE difference; compared with the ground-truth.

**Figure 9:**
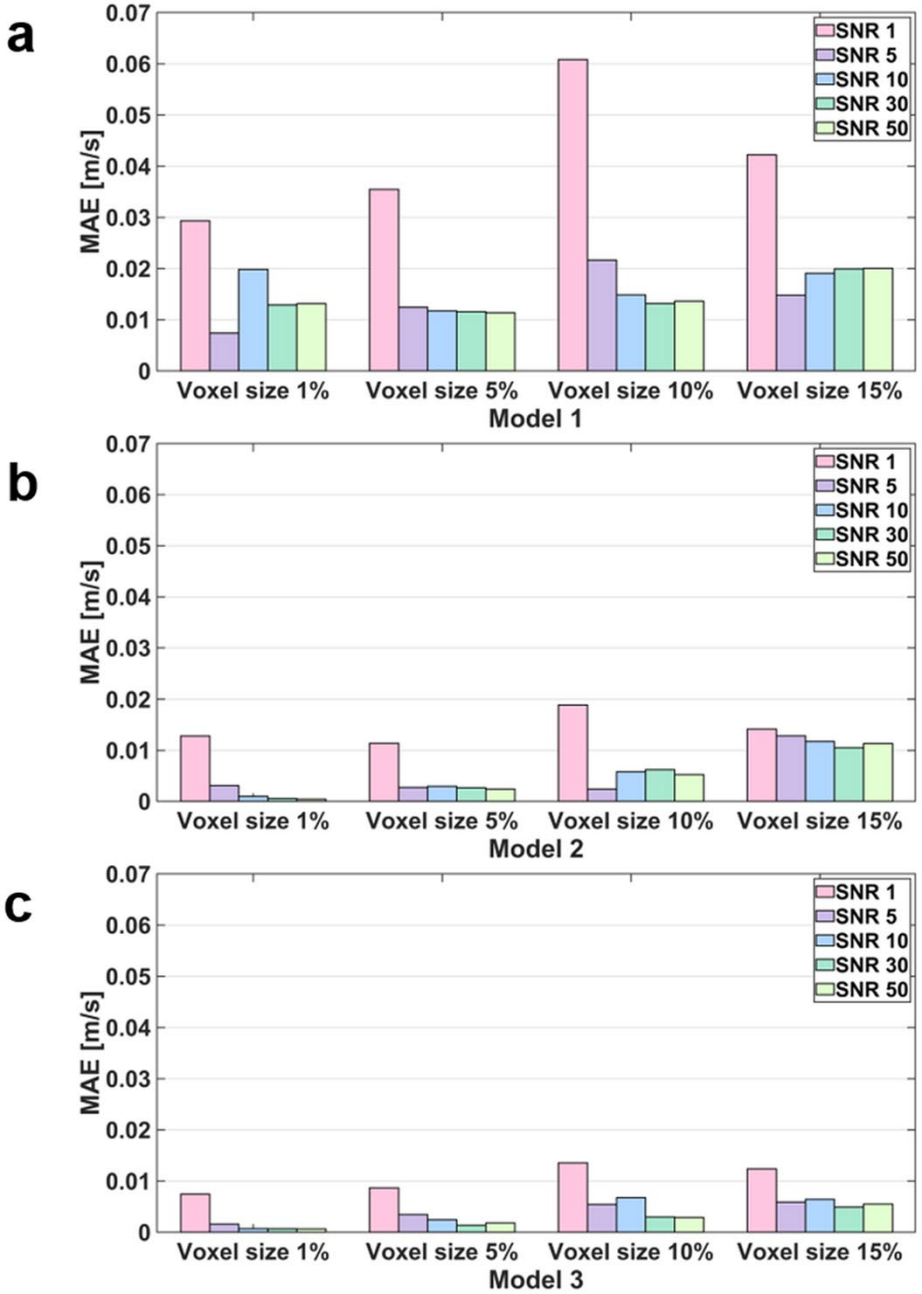
MAE of velocity magnitude for the TKE mapping inlet boundary condition with different noise and voxel size.

**Figure 10:**
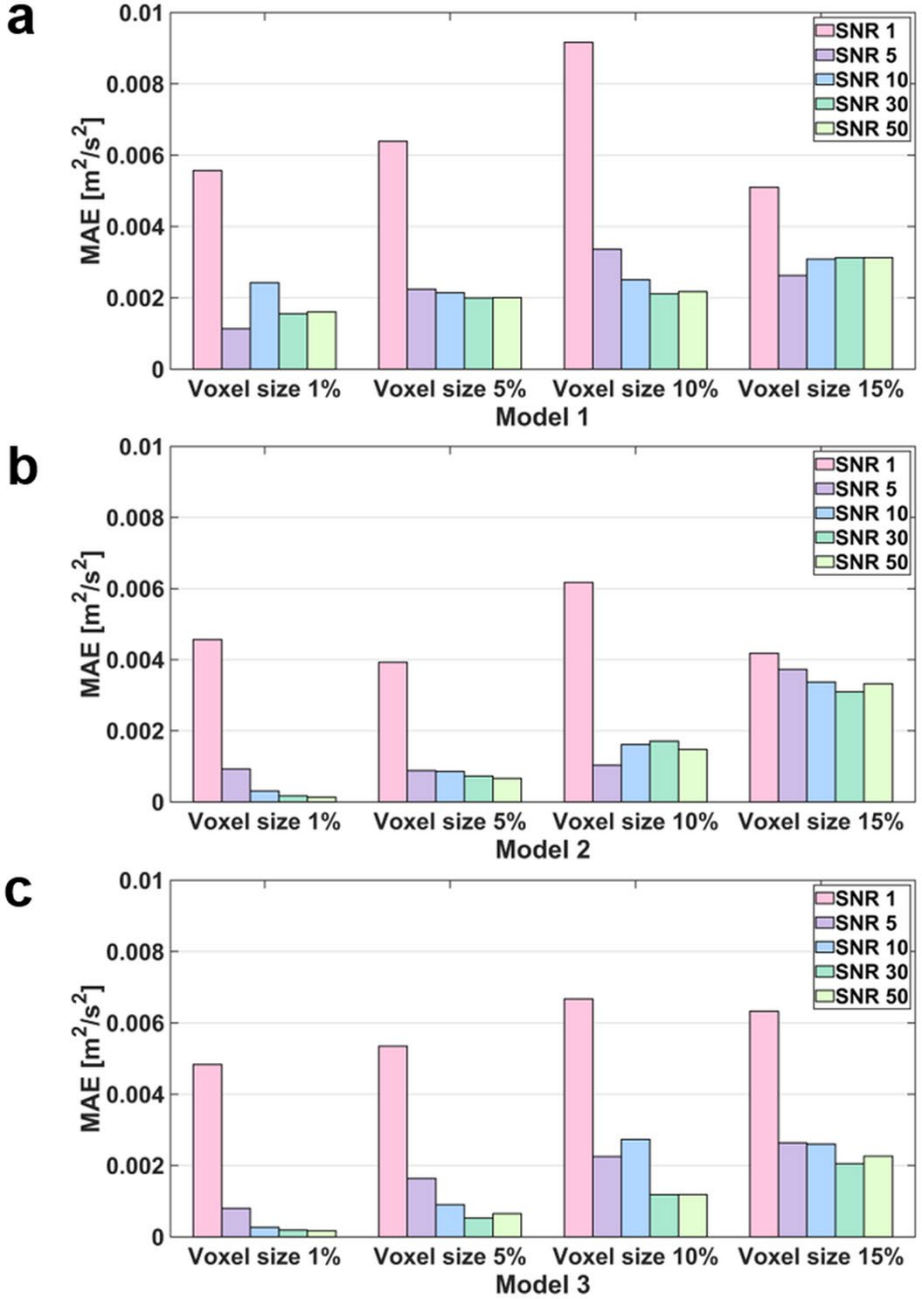
MAE of TKE for the TKE mapping inlet boundary condition with different noise and voxel size.

The flow simulations with the sparse TKE acquisition and overlapping noise were carried out to confirm the effect of experimental artifacts on the results. The results show that the errors of the velocity magnitude and TKE field increases as the signal-to-noise ratio (SNR) decreases and the voxel size increases (Figs. 7, 8, 9, 10). Among three different sub-models, Model 1 shows the highest discrepancy of the velocity and TKE regardless of the voxel size and SNR (Fig. 7 and 8). The highest MAE of the velocity and TKE are 0.061 m/s and 0.009 m^2^/s^2^ in Model 1, voxel size 10% and SNR 1 while the lowest MAE of the velocity is 0.0007 m/s in Model 3, voxel size 1% and SNR 50 and the lowest MAE of the TKE is 0.0002 m^2^/s^2^ in Model 2, voxel size 1% and SNR 50.

**Figure 11:**
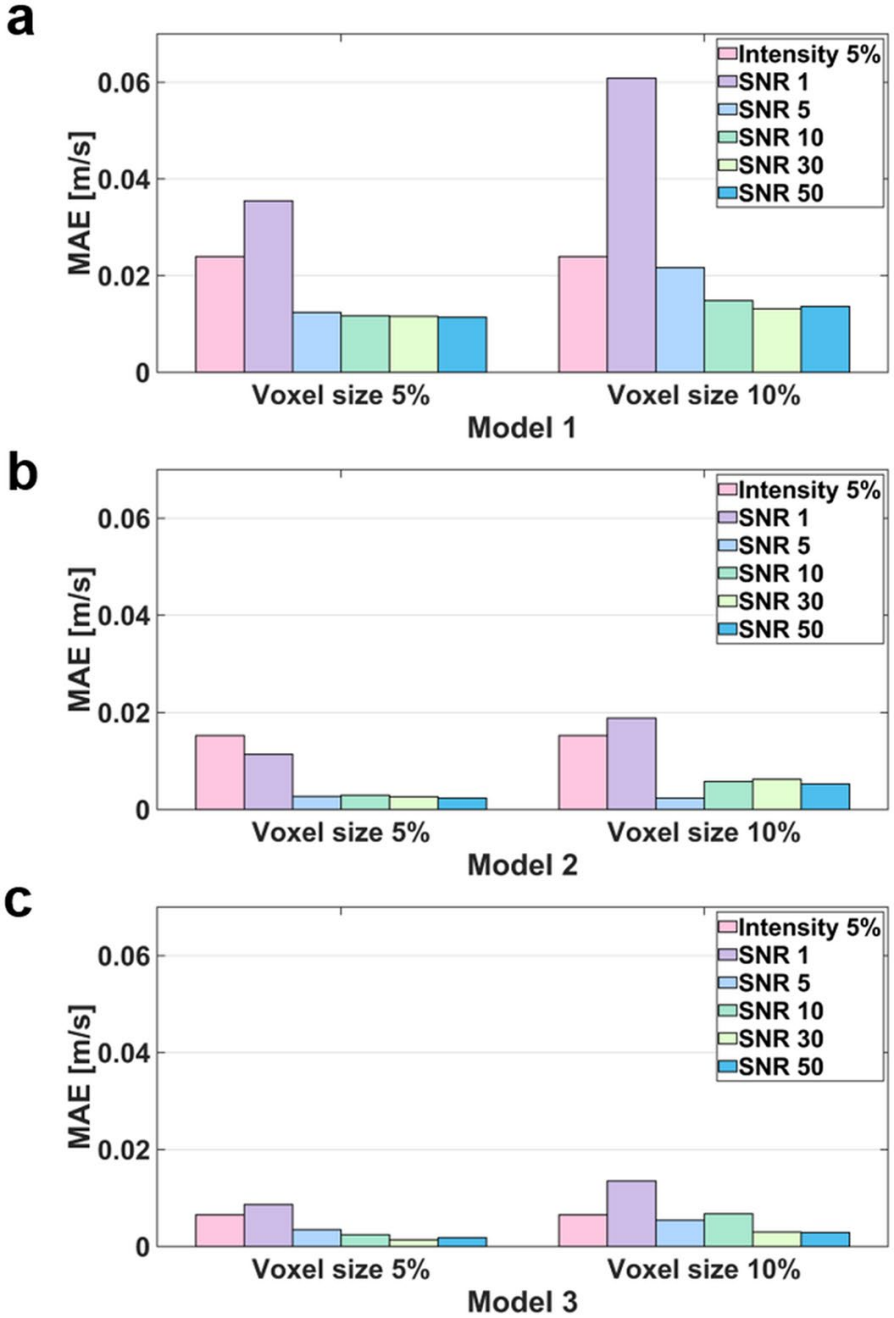
Comparison of velocity magnitude MAEs between turbulence intensity 5% and the TKE mapping inlet boundary conditions with different noise and voxel sizes.

**Figure 12:**
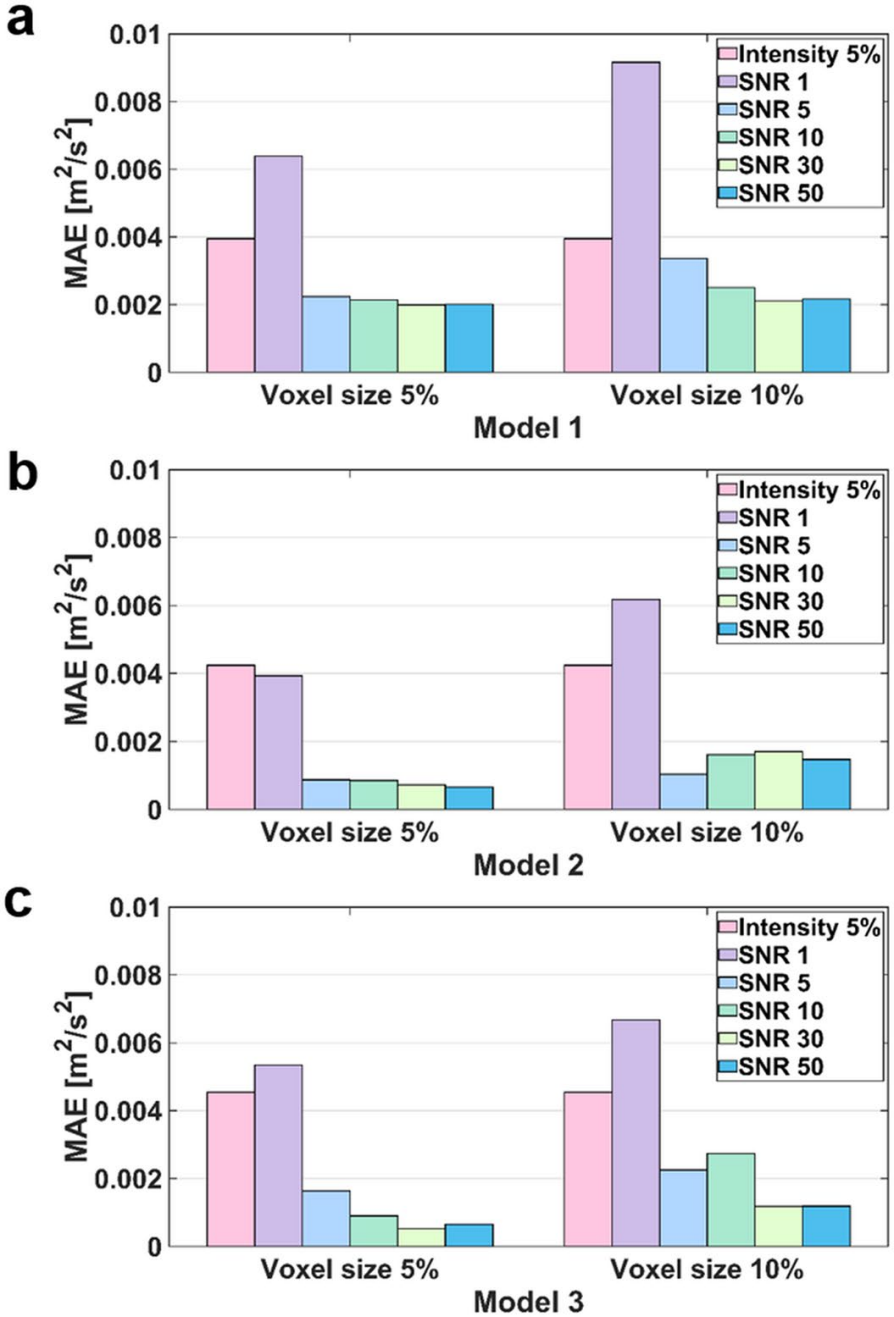
Comparison of TKE MAEs between turbulence intensity 5% and the TKE mapping inlet boundary conditions with different noise and voxel sizes.

The simulation results with arbitrary turbulence intensity and TKE boundary data measurements are compared (Fig. 11 and 12). For the voxel size of 5% and 10%, the simulations with TKE boundary data with SNR $\ge$ 5 resulted lower velocity and TKE errors than those with the turbulent intensity of 5%.

## Discussion

This study aimed to investigate the effect of turbulent boundary conditions on the accuracy of blood-flow simulations. The major findings of this study are as follows. (1) Although the successful selection of the turbulent intensity may provide the solution close to the ground-truth, inappropriate choice of turbulent intensity for the turbulent flow results large errors in the velocity and TKE distribution. (2) Directly providing TKE boundary data help simulating the turbulence flow, however, the accuracy of the simulation was affected by the TKE data quality. (3) The errors of the velocity magnitude and TKE field increased as the nose level increases and the TKE sampling is sparser. (4) For the practical measurement environment with voxel size of 5% to 10% and SNR $\ge$ 5, the simulations with given TKE boundary data resulted higher accuracy than those with the turbulent intensity.

Turbulent flow simulation requires turbulence quantities for the boundary condition. If the high-resolution measurement for the turbulent flow is available, the turbulent flow can be accurately expressed. However, the turbulence data are frequently lacking, especially for the blood flow. Alternatively, The turbulence description with the indirect parameters can be used such as turbulence intensity, turbulent viscosity ratio, hydraulic diameter, and turbulence length scale^38^. The turbulence intensity approach computes the inlet TKE boundary condition as follows:

$$\mathrm{k}=\frac{3}{2}{(UI)}^{2},$$

where U is the mean flow velocity and I is the turbulence intensity.

For internal flow, the turbulence intensity at the core of a fully-developed duct flow can be estimated from the following formula:

$$I=0.16{Re}_{{d}_{h}}^{-\frac{1}{8}},$$

where ${Re}_{{d}_{h}}$ is the Renolds number based on the pipe hydraulic diameter ${d}_{h}$^38^. Russo and Basse derived turbulence intensity scaling laws based on CFD simulations in smooth pipes and the turbulence intensity over the pipe area was defined as an arithmetic mean^39^. However, exact rule for the complex flow is not established.

The accurate choice of the turbulent intensity for the turbulent blood flow simulation is not straightforward. Conventional medical imaging such as Doppler echocardiography and phase-contrast MRI only provides peak velocity from the overlapping ultrasound signals or phase-averaged velocity from the multiple MRI echo signals^30,31^. Therefore, the previous studies on the turbulent blood flow usually assumed that the inlet flow is laminar or the turbulence intensity model is used with the choice of the turbulent intensity value^16,32,33^. Kimura et al., simulated the aortic blood flow without turbulence model even in patients with a stenotic bicuspid aortic valve^40^. Benim et al., simulated the blood flow in human aorta with a rather low turbulence intensity of 0.1%, which is close to laminar flow condition by neglecting the flow disturbance from the heart valve^41^. Perinajová et al., and Schubert et al., simulated turbulent flows mimicking a patient-specific coarctation of the aorta with a 5% turbulent intensity^7,42^. Moreover, some of the previous literature does not describe the turbulence inlet condition^43–45^.

The choice of the turbulent intensity is particularly important for the aortic blood flow simulation. Despite the phase-contrast MRI and 4D flow MRI provide 2D or 3D velocity of the blood flow, high spatio-temporal velocity data around the aortic valve leaflet with motion is usually limited. Therefore, many studies choose the inlet plane distal to the aortic valve level and simulate the downstream blood flows^46–48^. As the inlet plane locates distal to the aortic valve leaflet, the incoming flow is physiologically turbulent and its intensity various with the severity of the aortic valve stenosis^49^.

This study demonstrated the effect of the turbulent intensity boundary condition on the stenotic turbulent flow. Particularly, three sub-models were simulated to study three different inlet planes with different turbulent intensity. The results showed that the TKE field after stenosis was overestimated with the increase of turbulent intensity. The accuracy also varied with the sub-models even at the same turbulence intensity. This agrees with the previous study on the prediction of flow field in the FDA idea medical device with different turbulent inlet conditions^50^. In addition, it was confirmed that there were limitations in describing the TKE field near the inlet, which affected not only the nearby inlet but also downstream. Despite of the choice of turbulence intensity significantly affected the velocity and also TKE field, the optimal choice is not straightforward without preliminary knowledge on the flow field.

The advances of the medical imaging for the hemodynamics assessment such as 4D flow MRI now enables the direct quantification of the TKE field. Recently, the application of 4D flow MRI for turbulence estimation has been widely verified by detecting the changes in MRI signal magnitude according to spin distribution inside the voxel^49,51–53^. Previously, conventional 4D flow MRI sequence was used to estimate TKE, which is the trace of the Reynolds stress tensor (RST), for non-invasive measurement of turbulent in the aortic blood flow^53^. This 4D flow MRI sequence was further extended with a six-directional icosahedral (ICOSA6) flow-encoding scheme to measure all elements of RST, rather than only three diagonal elements, in turbulent flows^54–57^. Recently, full Reynolds stress quantification has been demonstrated for the normal subjects and the patients with the aortic stenosis^37^.

In this study, we investigated if applying patient-specific TKE data would work better than using an arbitrary turbulence intensity. While giving accurate TKE boundary data at the inlet should work better, the simulation accuracy with the experimental TKE data with limited resolution and noise is questioned. Despite of the limited resolution and noise level, the simulations with TKE boundary data with SNR $\ge$ 5 and the voxel size of 5% and 10% resulted lower velocity and TKE errors than those with the turbulent intensity of 5%. The reason is that even low-resolution and noisy data contain actual conditions. Furthermore, considering that the maximum blood flow velocity in the ascending aorta is approximately 1.0 m/s, an MAE of 0.05 m/s can be regarded as a significant error^58,59^. As mentioned above, turbulence intensity-based simulation is a method used when there is no turbulence quantity profile at the inlet, so it is natural that a larger error occurs compared to the simulation by the profile. Furthermore, in turbulent intensity-based simulation, if the turbulent boundary condition that is assigned initially is changed in the middle of iteration, an error may occur.

The practical applications of this study are as follows. (1) The results of this study can provide guidelines for obtaining accurate simulation results in studies using the RANS simulation method. (2) This study can provide a guide for developing a medical imaging device to improve the resolution of medical images, enabling a higher quality of simulation results. (3) This study can be combined with data assimilation studies that aim to complement the resolution of imaging devices in the future.

This study was conducted under conditions similar to those of previous studies; however, it has several limitations. First, blood was assumed to be a Newtonian fluid. Although non-Newtonian fluids using the Carreau-Yasuda viscosity model have been applied in various simulation studies, this study assumed a Newtonian fluid as the computation models were modeled in the same way as a blood vessel with a relatively large diameter, such as the aorta. The second limitation of this study lies in the selection of the k-epsilon model. This model was chosen due to its advantageous features, such as its widely validated nature among RANS models and its relative computational efficiency compared to other models. Various turbulence models, including LES, DNS, and RANS, can be employed for investigating blood flow dynamics. While recent research has focused on high-fidelity analysis methods such as LES, their application in clinical settings within actual hospital environments is constrained by computational costs and time requirements. The objective of this study was to explore the potential enhancement of CFD analysis by incorporating velocity and turbulence data obtained from 4D flow MRI measurements. To facilitate clinical implementation, we utilized the practical k-epsilon model, which is well-suited for hospital environments. Future studies aim to further validate our results by verifying the outcomes of other turbulence models. In addition, we conducted K-omega SST simulations under identical conditions to directly compare the results. A consistent trend of decreasing MAE with increasing SNR was observed, and similarly, higher MAE values were consistently evident at SNR50 compared to SNR30 (Fig. S2). With regards to the third limitation, a steady-state simulation was conducted. However, as the primary objective of this research is to investigate the impact of resolution and noise on simulations utilizing images, a steady-state simulation was preferred for this study. We plan to conduct a transient simulation to confirm our results in a patient model. The fourth limitation of this study is the absence of large eddy simulation (LES) or direct numerical simulation (DNS) results. Comparing the results of the Reynolds-averaged Navier–Stokes (RANS) model with LES or DNS results is a common practice due to the limitations of the RANS model in capturing turbulence. However, as the primary focus of this study was to examine the impact of modifying the inlet boundary conditions, the inclusion of LES and DNS results was not deemed necessary. Nevertheless, a grid system has been established to facilitate future investigations, and simulations using various turbulence models, including LES and DNS, will be conducted. The presence of boundary layers within our grid system can introduce errors when utilizing the k-epsilon turbulence model. Nonetheless, as previously stated, this issue does not significantly impact our study as we employed the same grid system to assess the simulation results concerning the inlet conditions. Finally, the simulations were conducted using an ideal model. In future investigations, we intend to perform simulation studies utilizing a precise IVUS-based patient model and subsequently compare the outcomes with those obtained from 4D flow MRI. Moreover, recent simulation studies have incorporated fluid–structure interactions to account for the behavior of blood vessels^60–62^. Although this approach entails increased computational costs compared to CFD alone, it is deemed necessary for attaining more realistic simulation outcomes.

The simulation results of applying the turbulence intensity boundary condition confirmed that the stronger the turbulence intensity, the higher the overall TKE field. In addition, the simulation with the TKE mapping boundary condition confirmed that the lower the SNR and lower the resolution, the greater the error in the simulation result. These major findings can provide guidelines for obtaining the optimal resolution level of medical images that can be adopted in CFD simulations. Furthermore, it can be used in medical-image-based data assimilation research.

## Methods

**Figure 13:**
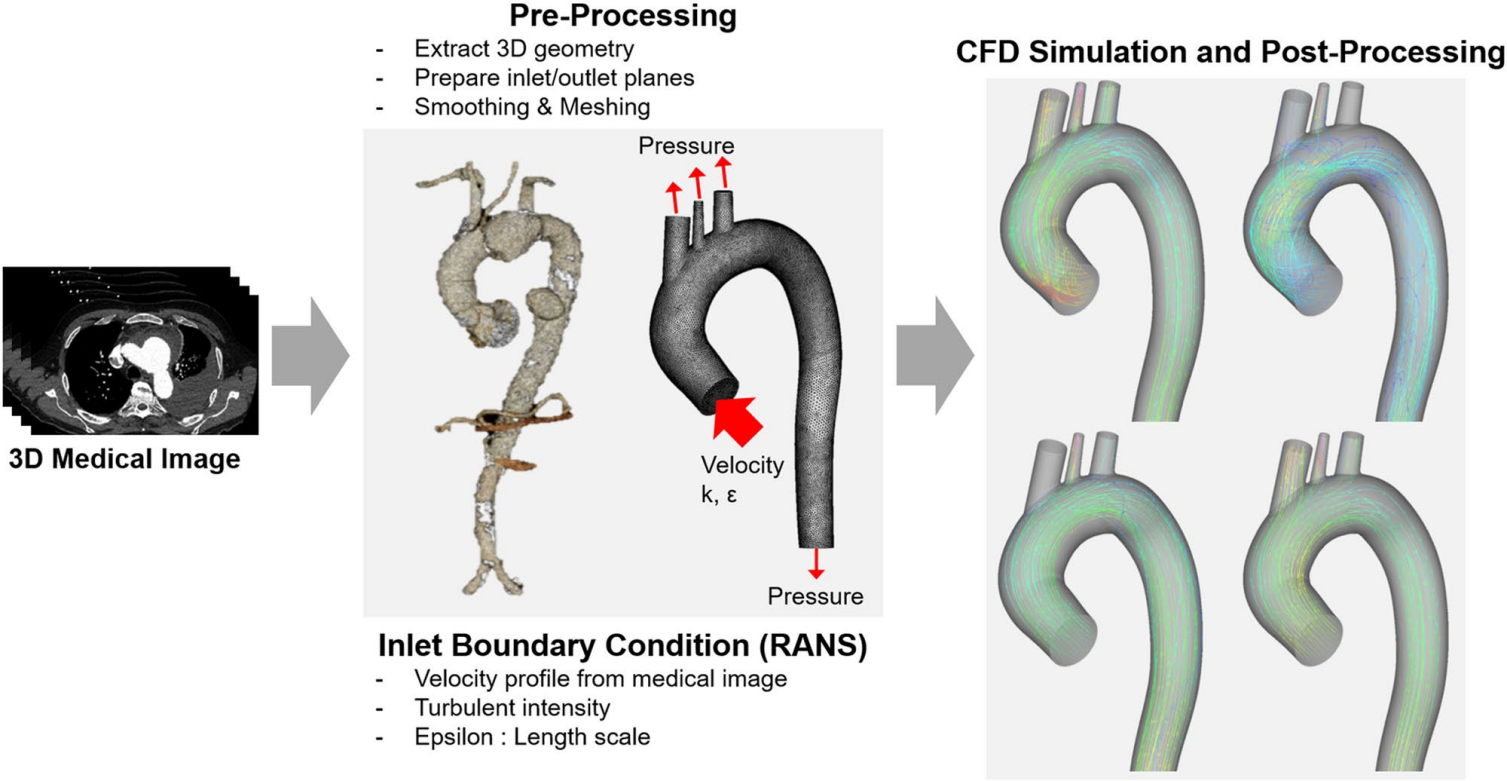
Schematic of turbulent blood flow simulation using the Reynolds-Averaged Navier–Stokes (RANS) model.

**Figure 14:**
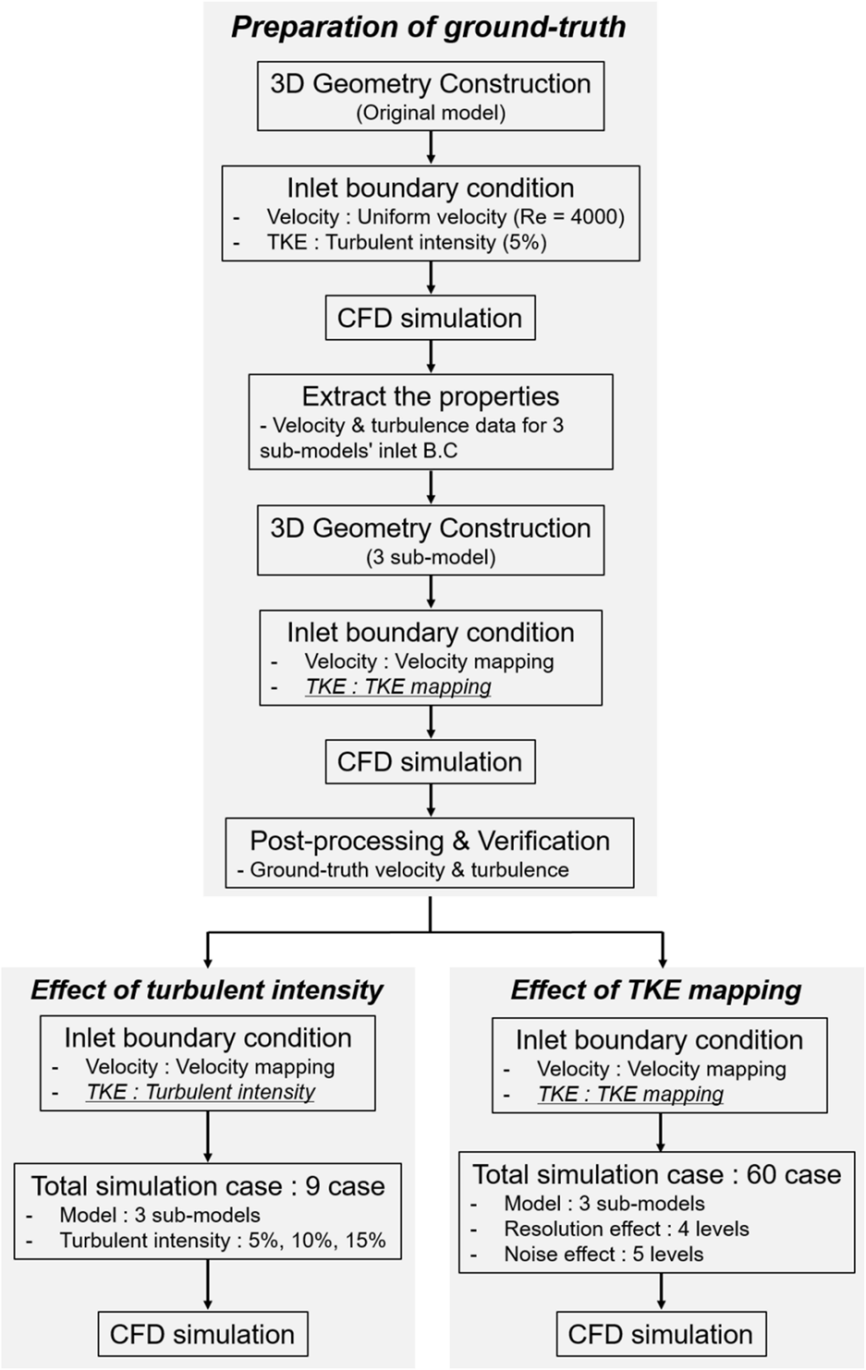
Workflow of the study to investigate the reproducibility of the computational fluid-dynamics (CFD) simulation at arbitrary turbulent intensity and TKE mapping with various resolution and noise levels.

As mentioned above, most of the RANS simulation uses the values calculated by modeling the turbulent inlet conditions (Fig. 13). In this study, we compare the simulation results using modeled turbulent inlet boundary conditions with the simulation results directly applying the turbulent kinetic energy field extracted from the medical image. Therefore, we constructed a simulation procedure and conducted simulations and comparisons based on it (Fig. 14).

### 3D model description

**Figure 15:**
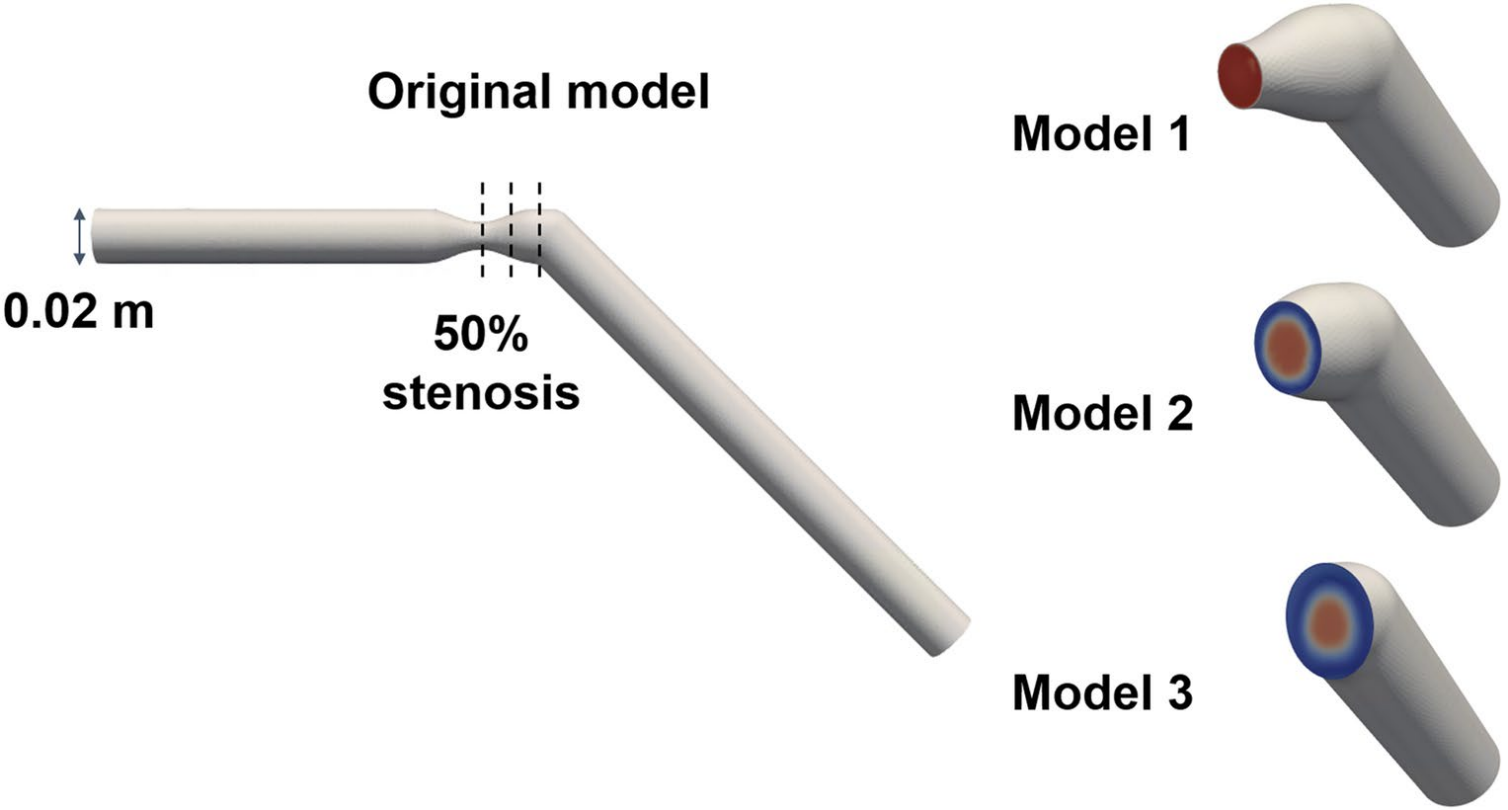
Model description; Original model and 3 sub-models. Model 1, 2, 3 were cut at the stenotic apex level, 0.5D and 1.0D distal to the stenosis.

A stenosis model with 50% severity in diameter was used to mimic the aortic blood flow with the heart valve (Fig. 15). The original full model had a diameter D of 0.02 m. Stenosis develops 6D downstream from the entrance. The length of the stenosis part was 2D. The models were bent at 45° to mimic the impinging aortic valvular flow at the ascending aorta. Three different planes at the stenosis apex, 0.5D and 1D distal from the stenosis apex were set to extract the sub-models, which have the different levels of turbulent inlet flow.

### Preparation of ground truth

The original full-model was simulated to obtain the ground truth. In our methodology, we initially conducted simulations using the original full-model, which encompassed the complete geometry. Subsequently, we utilized the results obtained from the simulation with the original full-model to extract velocity and TKE information from a specific plane that corresponded to the boundary plane for the sub-models, which included a partial geometry rather than the complete geometry. Furthermore, by employing the velocity and TKE information obtained from the aforementioned plane as the inlet boundary condition, we performed simulations on the sub-model without altering the noise level or resolution. The results obtained from this simulation were considered as the ground-truth reference. Finally, we compared the results of the simulation with the sub-model to those of the original full-model in order to determine whether the simulation with the sub-model could be used as a valid ground-truth representation or not. The uniform inlet velocity of 0.66 m/s was specified, which corresponds to the Reynolds number of 4,000 based on the inlet unconstructed area. The Reynolds number was set to mimic the physiological aortic blood flow^63^. The TKE boundary condition was applied using a turbulent intensity of 5%, and the energy-dissipation boundary condition was selected using a mixing length scale of 0.0014^38,64^. From the full-model simulation, the cross-sectional inlet velocity and TKE at three different planes were extracted for sub-model simulations. The mixing length scale was set as 0.000375^64^. The mixing length scale of the original full-model is based on the mixing layer flow and sub-models are based on the jet flow.

### Computational fluid dynamics and numerical scheme

This study employed the RANS equation based on the continuity, momentum, and transport equations. The time-averaged continuity and momentum equations are expressed by incorporating Einstein notation, as follows:

1 $$\frac{\partial {U}_{i}}{\partial {x}_{i}}=0,$$

2 $$\rho {U}_{j}\frac{\partial {U}_{i}}{\partial {x}_{j}}=\frac{\partial }{\partial {x}_{j}}\left[-P{\delta }_{ij}+2\mu {S}_{ij}-\rho \overline{{u}_{i}{u}_{j}}\right]+\rho \overline{{f}_{i}},$$

where ${U}_{j}$, $P$, $\rho$, $\mu$, and $\overline{{f}_{i}}$ denote the velocity, pressure, density, dynamic viscosity, and external force, respectively. ${\delta }_{ij}$ is the Kronecker delta, and ${S}_{ij}$ is the rate of the mean strain tensor, expressed as ${S}_{ij}\equiv 1/2\left[\partial {U}_{i}/\partial {x}_{j}+\partial {U}_{j}/\partial {x}_{i}\right]$. Blood is assumed to be an incompressible Newtonian fluid. The fluid properties of density and dynamic viscosity for the representation of blood were set to 1060 kg/m^2^ and 3.5 cP, respectively^65,66^.

This study employed the standard k–ε model as a turbulence model. The gradient terms were discretized using a second-order Gaussian central differencing scheme. The divergence terms for the momentum equation were discretized using a second-order upwind scheme with a bounded option, and for the turbulence equation, a limited linear scheme with a bounded option was used. The simulation converged to a normalized residual of 1.0e-4 for all the physical quantities. The wall condition was assumed to be a rigid body with a no-slip boundary condition, and pressure was uniformly applied zero on the outlet boundary condition.

A numerical simulation was performed using the open-source finite volume method software OpenFOAM. The computational domains for the fluid were meshed using Fluent (Ansys, Inc., PA, USA). The first layer thickness of 0.01 mm and a total of 15 boundary layers with a growth rate of 20% were used to obtain ${y}^{+}<1$, here ${y}^{+}$ is the wall distance, expressed as ${y}^{+}\equiv {u}_{\tau }y/\nu$, $y$ is the exact normal distance from a solid surface, ${u}_{\tau }$ is the friction velocity, and $\nu$ is the kinematic viscosity.

In this study, 1,650,832 tetrahedral elements for the original model were used to discretize the geometry. The grid independence test results can be found as Supplementary Table S1 and Figure S1^67^. Maximum velocity is a very important factor in clinical surgical guidelines, so we conducted a grid test based on it^68^. Maximum TKE is also used because of its clinical importance in the literature^36,49,52,53^. The number of tetrahedral elements for three sub-models (Model 1, Model 2 and Model 3) are 1,038,263, 1,016,577 and 977,318, respectively.

### The effect of turbulent intensity

To investigate the effect of turbulent intensity on the inlet boundary condition, nine different simulations were conducted with three levels of turbulence intensity and three sub-models. For a statistical comparison with the ground truth, the MAE is calculated as follows:

3 $$\mathrm{MAE}=\sum_{i=1}^{n}\frac{\left|{V}_{i}^{Sub}-{V}_{i}^{Ori}\right|}{n},$$

where ${V}_{i}^{Ori}$ and ${V}_{i}^{Sub}$ are the properties of the original and sub-models on $i$-th node, respectively.

### The effect of the noise and resolution of the TKE boundary data

**Figure 16:**
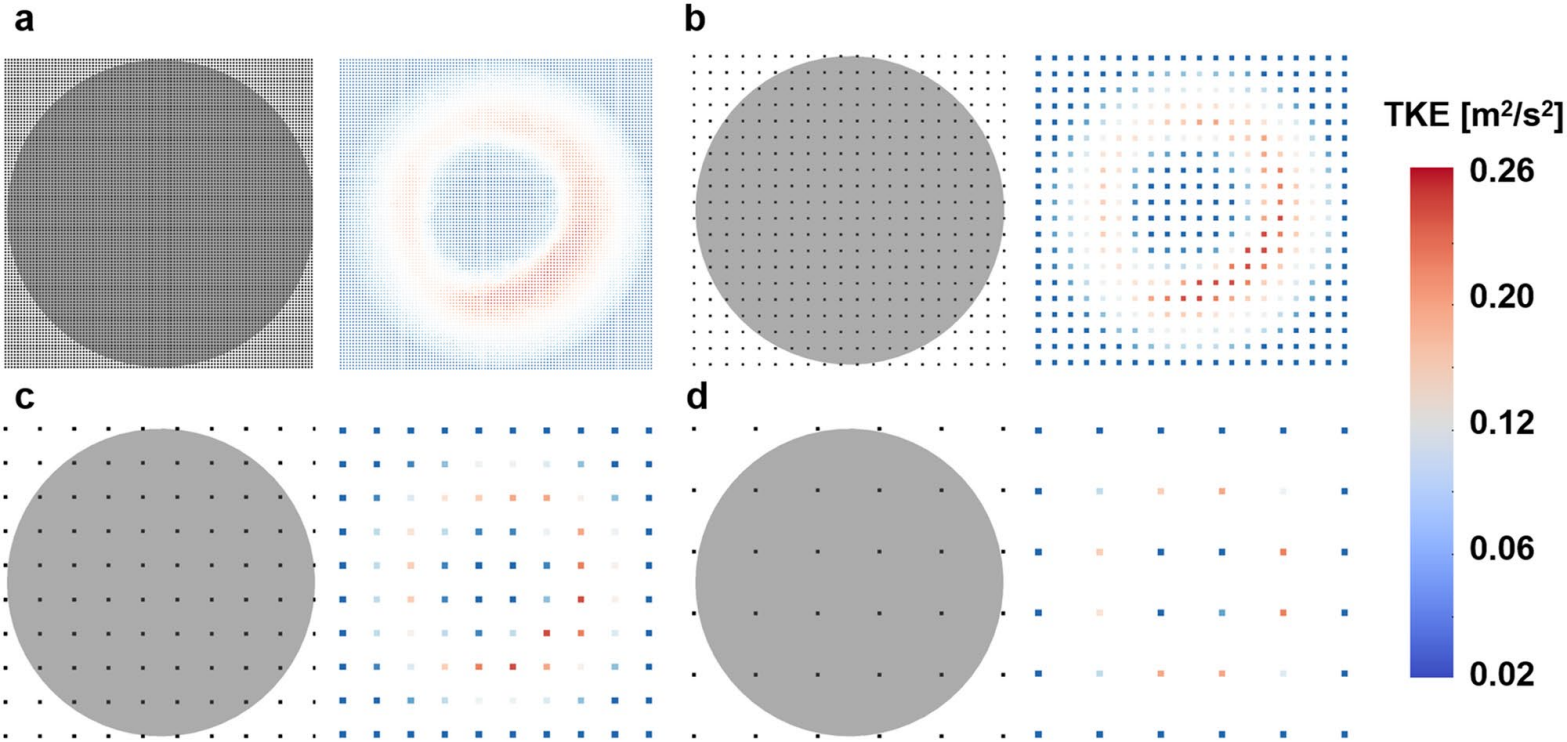
TKE sampling for simulating the experimental measurement; (**a**) voxel size 1%, (**b**) voxel size 5%, (**c**) voxel size 10%, (**d**) voxel size 15%. The voxel size is ratio of lumen diameter and distance between pixels. Left and right panels indicate the measurement resolution compared to the inlet plane and corresponding TKE distribution.

To confirm the effect of the noise and resolution of TKE boundary data on the simulation results, a total of 60 simulations were conducted with different noise and resolution levels. The part of the TKE boundary data were sampled according to the resolution levels (Fig. 16). The four different level of resolution was set to 1%, 5%, 10%, and 15%, which is the ratio of the voxel sampling size to the original diameter size. A total of five different SNR^1,5,10,30,50^ was generated by adding Gaussian noise, where SNR is defined to the maximum TKE to the standard deviation of the noise.

### Supplementary Information

Supplementary Information.

## Data Availability

The datasets used and/or analyzed during the current study are available from the corresponding author upon reasonable request.
