# Supplementary material for: Assessing the impact of turbulent kinetic energy boundary conditions on turbulent flow simulations using computational fluid dynamics
Source: Sci Rep. 2023 Sep 5;13:14638. doi: 10.1038/s41598-023-41324-w (PMC10480182; doi:10.1038/s41598-023-41324-w)
Supplement: Supplementary file 1 — Supplementary Information. [file 41598_2023_41324_MOESM1_ESM.docx]

**Assessing the impact of turbulent kinetic energy boundary conditions on turbulent flow simulations using computational fluid dynamics**

Eui Cheol Jung^1^, Gyu-Han Lee^2^, Eun Bo Shim^3^, Hojin Ha^3^

^1^ Institute of Inclusive Technology, Kangwon National University, 1, Kangwondaehak-gil, Chuncheon 24341, Republic of Korea.

^2^ Department of Interdisciplinary Program in Biohealth-Machinery Convergence Engineering, Kangwon National University, 1, Kangwondaehak-gil, Chuncheon 24341, Republic of Korea

^3^ Department of Mechanical and Biomedical Engineering, Kangwon National University, 1, Kangwondaehak-gil, Chuncheon 24341, Republic of Korea

**Corresponding Author**: Hojin Ha

Department of Mechanical and Biomedical Engineering,

Kangwon National University,

1, Kangwondaehak-gil, Chuncheon 24341, Republic of Korea.

Phone: +82-33-250-6318

Fax: +82-33-257-6595

E-mail: hojinha@kangwon.ac.kr

**Table S1** Calculations of discretization error.

|  | $\emptyset$=Maximum velocity at stenosis apex | $\emptyset$=Maximum TKE at stenosis apex |
| --- | --- | --- |
| N_1_, N_2_, N_3_ (millions) | 3.17, 1.65, 1.15 | 3.17, 1.65, 1.15 |
| r_21_ | 1.943 | 1.943 |
| r_32_ | 1.426 | 1.426 |
| $\emptyset_{1}$ | 2.8065 | 0.0944 |
| $\emptyset_{2}$ | 2.8153 | 0.0936 |
| $\emptyset_{3}$ | 2.8300 | 0.0923 |
| p | 2.361 | 2.554 |
| $\emptyset_{ext}^{21}$ | 2.804 | 0.095 |
| $e_{a}^{21}$ | 0.31% | 0.80% |
| $e_{ext}^{21}$ | 0.08% | 0.18% |
| ${GCI}_{fine}^{21}$ | 0.10% | 0.22% |
|  | $\emptyset$=Maximum velocity at 0.5D distal from stenosis apex | $\emptyset$=Maximum TKE at 0.5D distal from stenosis apex |
| N_1_, N_2_, N_3_ (millions) | 3.17, 1.65, 1.15 | 3.17, 1.65, 1.15 |
| r_21_ | 1.943 | 1.943 |
| r_32_ | 1.426 | 1.426 |
| $\emptyset_{1}$ | 2.4279 | 0.2084 |
| $\emptyset_{2}$ | 2.4312 | 0.1846 |
| $\emptyset_{3}$ | 2.4267 | 0.1834 |
| p | 0.632 | 2.683 |
| $\emptyset_{ext}^{21}$ | 2.422 | 0.213 |
| $e_{a}^{21}$ | 0.14% | 11.38% |
| $e_{ext}^{21}$ | 0.26% | 2.25% |
| ${GCI}_{fine}^{21}$ | 0.33% | 2.88% |
|  | $\emptyset$=Maximum velocity at 1.0D distal from stenosis apex | $\emptyset$=Maximum TKE at 1.0D distal from stenosis apex |
| N_1_, N_2_, N_3_ (millions) | 3.17, 1.65, 1.15 | 3.17, 1.65, 1.15 |
| r_21_ | 1.943 | 1.943 |
| r_32_ | 1.426 | 1.426 |
| $\emptyset_{1}$ | 2.3178 | 0.2650 |
| $\emptyset_{2}$ | 2.3141 | 0.2488 |
| $\emptyset_{3}$ | 2.3168 | 0.2340 |
| p | 0.370 | 1.078 |
| $\emptyset_{ext}^{21}$ | 2.330 | 0.281 |
| $e_{a}^{21}$ | 0.16% | 6.12% |
| $e_{ext}^{21}$ | 0.53% | 5.53% |
| ${GCI}_{fine}^{21}$ | 0.67% | 7.31% |

To assess the mesh independence, we conducted simulations using three distinct meshes and compared the resulting maximum velocity values. As the objective of this study revolves around characterizing the inlet boundary conditions, the maximum velocity and maximum turbulent kinetic energy were determined for each inlet cross section. Maximum velocity is a very important factor in clinical surgical guidelines, so we conducted a grid test based on it. Maximum TKE is also used because of its clinical importance in the literature.

**Fig S1.** The velocity and turbulent kinetic energy (TKE) results along the y-direction were analyzed and obtained at the stenosis apex (Z=0)


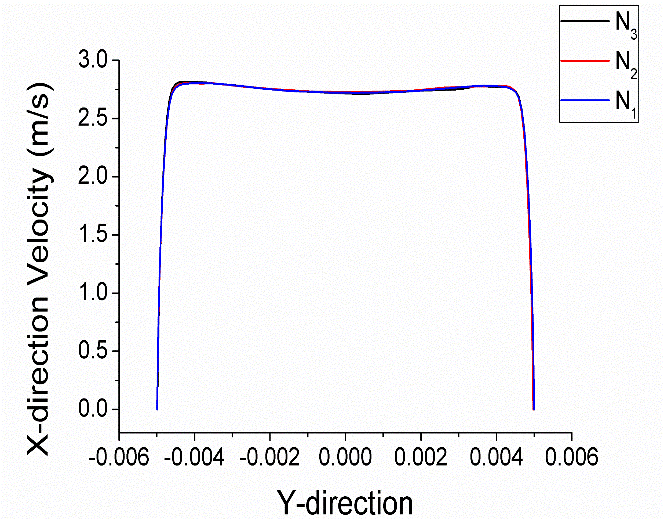

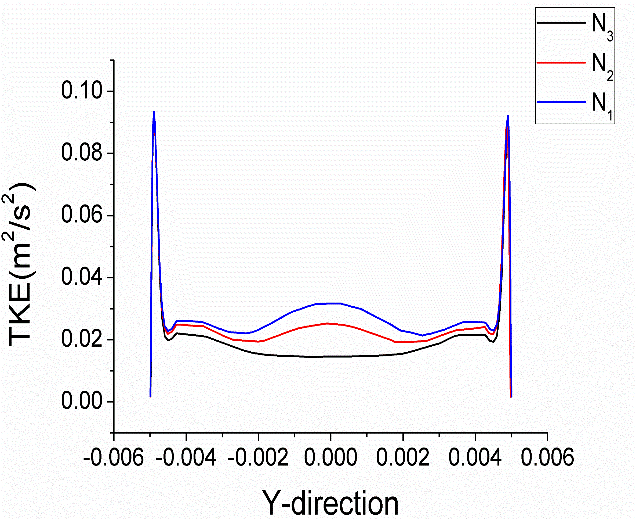


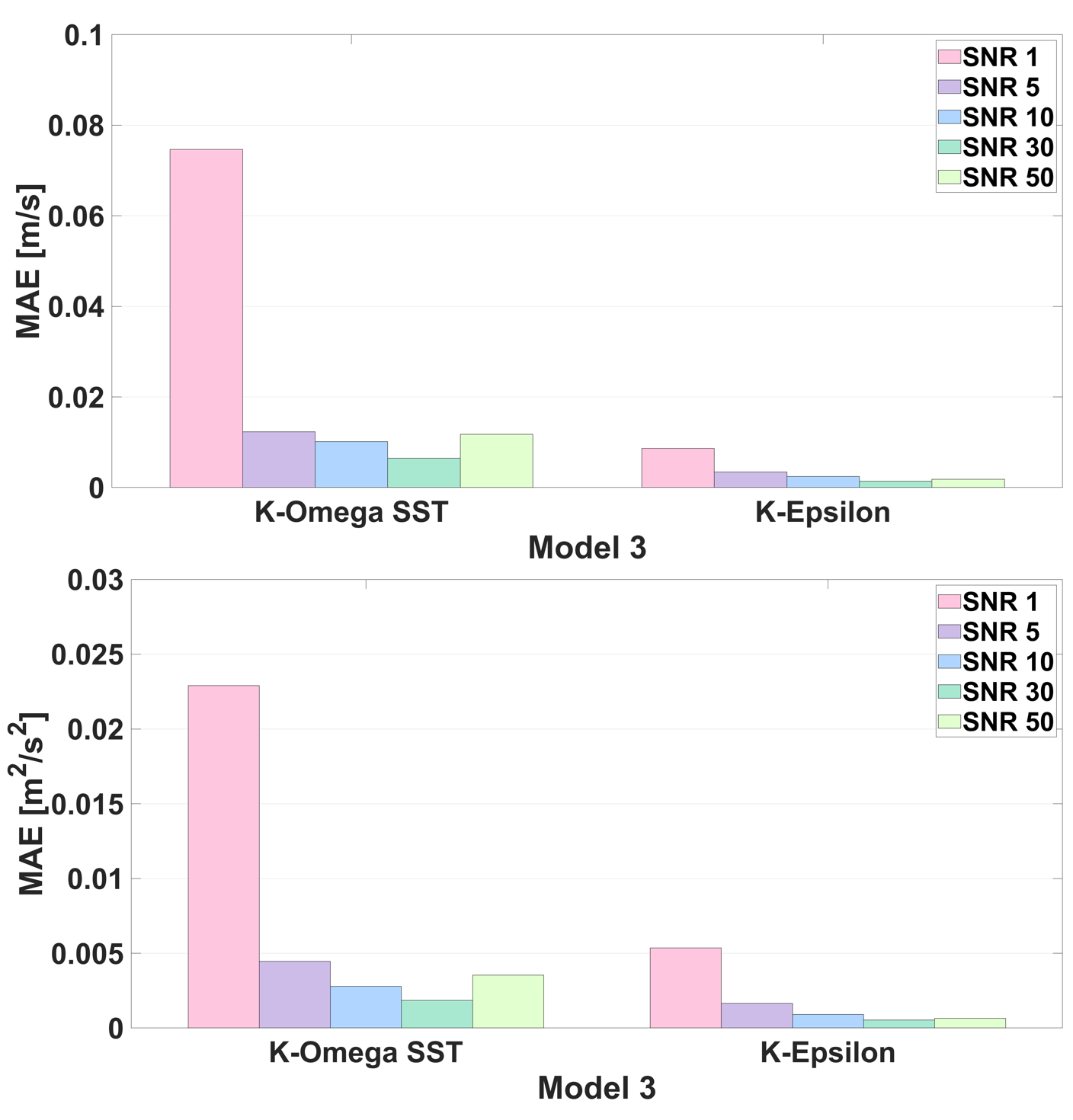


**Fig S2.** Comparison of simulation results using the K-omega SST and K-epsilon turbulence models
